# Using thermal scanning assays to test protein-protein interactions of inner-ear cadherins

**DOI:** 10.1371/journal.pone.0189546

**Published:** 2017-12-19

**Authors:** Deepanshu Choudhary, Anusha Kumar, Thomas J. Magliery, Marcos Sotomayor

**Affiliations:** Department of Chemistry and Biochemistry, The Ohio State University, Columbus, Ohio, United States of America; University of Illinois at Chicago, UNITED STATES

## Abstract

Protein-protein interactions play a crucial role in biological processes such as cell-cell adhesion, immune system-pathogen interactions, and sensory perception. Understanding the structural determinants of protein-protein complex formation and obtaining quantitative estimates of their dissociation constant (*K*_D_) are essential for the study of these interactions and for the discovery of new therapeutics. At the same time, it is equally important to characterize protein-protein interactions in a high-throughput fashion. Here, we use a modified thermal scanning assay to test interactions of wild type (WT) and mutant variants of N-terminal fragments (EC1+2) of cadherin-23 and protocadherin-15, two proteins essential for inner-ear mechanotransduction. An environmentally sensitive fluorescent dye (SYPRO orange) is used to monitor melting temperature (*T*_m_) shifts of protocadherin-15 EC1+2 (pcdh15) in the presence of increasing concentrations of cadherin-23 EC1+2 (cdh23). These *T*_m_ shifts are absent when we use proteins containing deafness-related missense mutations known to disrupt cdh23 binding to pcdh15, and are increased for some rationally designed mutants expected to enhance binding. In addition, surface plasmon resonance binding experiments were used to test if the *T*_m_ shifts correlated with changes in binding affinity. We used this approach to find a double mutation (cdh23(T15E)- pcdh15(G16D)) that enhances binding affinity of the cadherin complex by 1.98 kJ/mol, roughly two-fold that of the WT complex. We suggest that the thermal scanning methodology can be used in high-throughput format to quickly compare binding affinities (*K*_D_ from nM up to 100 μM) for some heterodimeric protein complexes and to screen small molecule libraries to find protein-protein interaction inhibitors and enhancers.

## Introduction

Protein-protein interactions are intrinsic to virtually every cellular process whether it be transcription, translation, replication, cell cycle control or signal transduction [1–3]. These interactions are also essential for multicellular organisms in processes such as cell-cell adhesion, host-pathogen interactions, and sensory perception [4–6]. Measurements of binding affinity and kinetic rate constants are often used along with site-directed mutagenesis to understand the molecular mechanisms underlying protein-protein interactions. The quantitative determination of dissociation constants (*K*_D_) can be done in multiple ways, e.g., by measuring association-dissociation kinetics or by determining the fraction of complex formed as a function of “protein ligand” concentrations. These measurements often involve detection of heat (isothermal calorimetry) or optical signals (surface plasmon resonance, fluorescence anisotropy, and analytical ultracentrifugation), and in some cases require large amounts of samples or labeling that could perturb the binding interaction [7–13]. Most of these methodologies cannot be easily used in a high-throughput fashion to test the effect of mutations as well as various ligands and buffers.

Thermal stability shift assays (also referred to as differential scanning fluorimetry or high-throughput thermal scanning when combined with high-throughput purification) have been commonly used to optimize the thermal stability of a single protein or multiple protein variants under different conditions [14–25], but have not been widely used to test protein-protein interactions. Recently, thermal scanning was used to characterize and quantify interactions in a heterodimeric protein complex [26]. In this approach, samples of a protein (maltose-binding protein, MBP) with low melting temperature, *T*_m1_, were mixed in non-stoichiometric amounts with an analyte ligand (synthetic ankyrin repeat protein, Off7) with higher melting temperature (*T*_m2_ > *T*_m1_) in the presence of the SYPRO orange dye [27]. The melting temperatures of the MBP protein alone and when mixed with the “analyte” were measured by monitoring the increased fluorescence of the dye upon binding to hydrophobic portions of the unfolded proteins. Binding of a protein to the folded state of its partner was found to increase its apparent melting temperature, and was used to quantify the binding affinity of wild-type (WT) and mutant variants of the MBP/Off7 pair [26].

**Fig 1:**
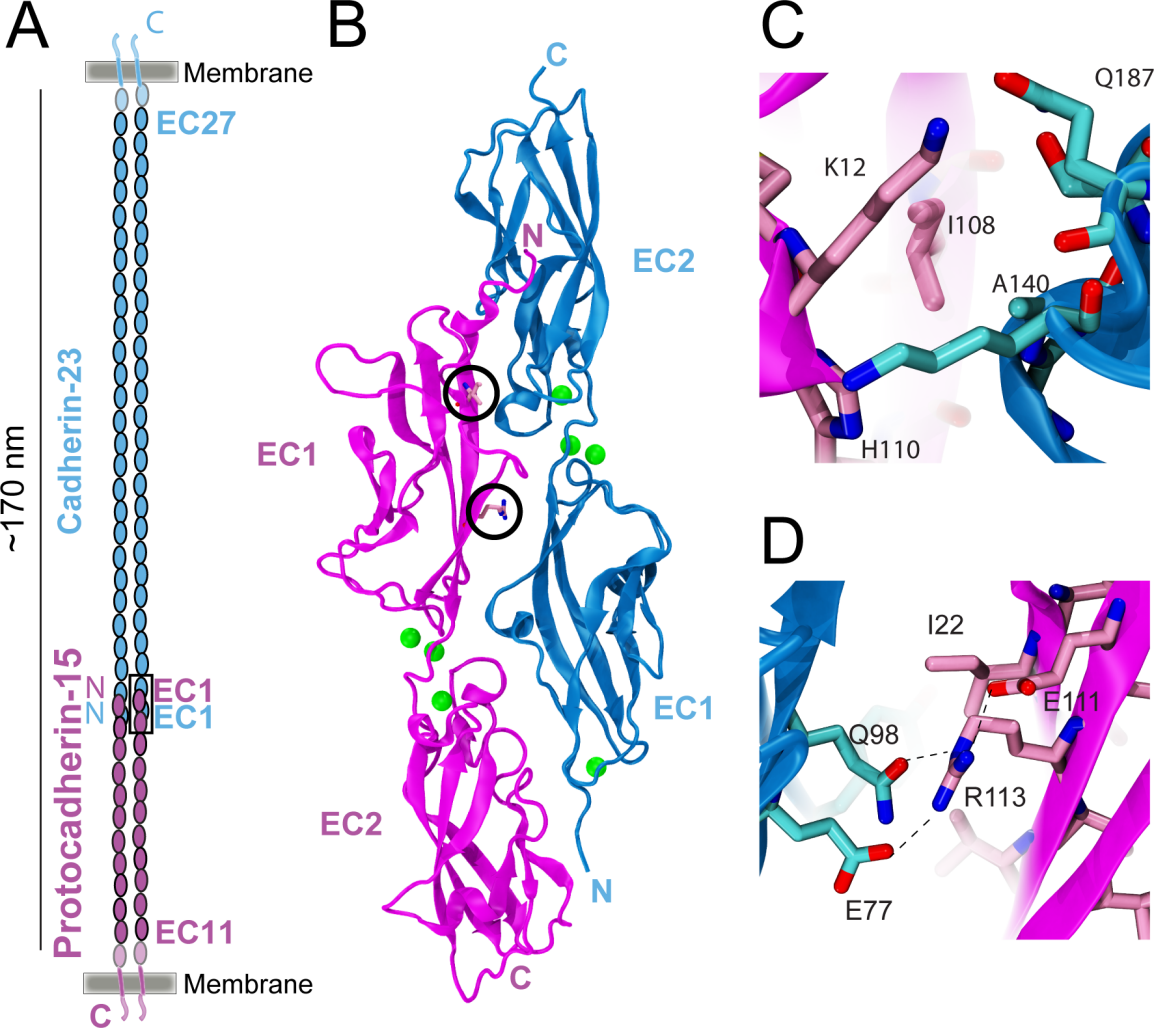
Heterotetrameric tip link made of CDH23 bound to PCDH15. (A) The tip link is formed by a CDH23 parallel dimer interacting tip-to-tip with a PCDH15 parallel dimer [39]. These proteins feature 27 and 11 extracellular cadherin (EC) repeats, respectively. (B) Ribbon diagram of mouse cdh23 (blue) bound to pcdh15 (magenta) with Ca^2+^ ions as green spheres (PDB ID: 4APX). Sites of deafness-causing mutations R113 and I108 in PCDH15 are shown in stick representation and circled. (C & D) Detail of I108 (C) and R113 (D) with surrounding residues in the cdh23 and pcdh15 interface.

Here, we applied a similar thermal scanning assay to a protein complex formed by two members of the cadherin superfamily, which consists of transmembrane proteins that often mediate calcium-dependent cell–cell adhesion in animals [4,28–31]. Cadherin-23 (CDH23) and protocadherin-15 (PCDH15) interact tip-to-tip (Fig 1A) to form “tip-link” filaments essential for inner-ear mechanotransduction [5,32–34]. CDH23 and PCDH15 have 27 and 11 extracellular cadherin (EC) repeats, respectively. Each EC repeat is made of about 100 amino acids, which are similar but not identical to each other in sequence and structure. The X-ray crystal structure of the mouse protein complex showed a heterophilic interaction that resembled an “extended handshake” and that involved the N-terminal EC1 and EC2 repeats from both proteins (Fig 1B). Complementary *in vitro* and *in vivo* studies revealed that two deafness-related missense mutations in PCDH15 (I108N and R113G; Fig 1C and 1D) severely disrupt binding of CDH23 EC1+2 (hereafter cdh23) to PCDH15 EC1+2 (pcdh15) [34–40].

Using thermal scanning with SYPRO orange to detect unfolding, we found that pcdh15 melts at lower temperatures than cdh23, and that the apparent melting temperature of pcdh15 increases in the presence of cdh23. This shift in melting temperature is absent when the cdh23-pcdh15 interaction is impaired by deafness mutations, and it increases for some rationally designed mutations expected to increase binding affinity of this complex. Furthermore, our surface plasmon resonance (SPR) experiments revealed that some of these designed mutations altered the dissociation rate constant (*k*_off_), while a double mutant (cdh23(T15E)-pcdh15(G16D)) increased binding stability by 1.98 KJ/mol, which corresponds to roughly twice the binding affinity of WT. Overall, our study highlights the benefits of combining rational design with a semi-quantitative high-throughput assay to study protein-protein interactions in heterodimeric complexes.

## Materials and methods

### Cloning and mutagenesis

Cloning and expression of 6xHis tagged protein fragments of mouse cdh23 and pcdh15 were previously described in [39,41]. Briefly, DNA sequences coding for both protein fragments were cloned using the NdeI and XhoI sites of the pET21a vector that includes a C-terminal His tag. All mutants used in this work were generated using the QuikChange Lightning mutagenesis kit (Stratagene) and were verified by DNA sequencing. Numbering of residues corresponds to mouse CDH23 and PCDH15 without their signal sequences.

Fragments used in SPR experiments were derived from a template provided by Dr. Yoshie Narui. Briefly, a DNA construct containing the N-terminal amino acid residues 1–205 (EC1+EC2) of mouse CDH23 was obtained by PCR amplification and subcloned using the Impact kit (NEB #E6901S) and the NdeI and SapI restriction sites of the pTXB1 vector (NEB #N6707). The pTXB1 attached the *Mycobacterium xenopi* GyrA intein tag at the C terminus of EC2. The tag contains an N-terminal cysteine residue that allows thiol-induced cleavage. Each intein tag contains a chitin-binding domain (CBD) for affinity purification of the fusion protein on a chitin resin. Induction of on-column cleavage, using thiol reagents such as dithiothreitol (DTT), releases the "tagless" cdh23 from the intein tag. An Ala residue was included between CDH23 EC2 and the intein tag to improve cleavage efficiency.

### Expression, purification, and refolding of protein fragments

All cdh23 fragments with a His tag were expressed independently in BL21(DE3)-pLysS *E*. *coli* cells (Stratagene) cultured in lysogeny broth (LB) or terrific broth (TB) medium and induced at OD_600_ ~ 0.6 with 1 mM IPTG at 30°C for ~ 16 h. All pcdh15 fragments were expressed independently in BL21CodonPlus(DE3)-RIPL *E*. *coli* cells (Stratagene) cultured in TB medium and induced at OD_600_ ~ 0.6 with 200 μM IPTG at 30°C for ~ 16 h. Cells were lysed by sonication for 7 min in denaturing buffer B (20 mM Tris-HCl at pH 7.5, 6 M guanidine hydrochloride [GuHCl], 10 mM CaCl_2_, 20 mM imidazole at pH 7.0). Cell lysate was collected after centrifugation at 4°C. The clear lysates were loaded onto nickel-sepharose beads (GE Healthcare) and eluted with denaturing buffer B supplemented with 500 mM imidazole (buffer E) after thorough washing with denaturing buffer B. The refolding of WT and mutant pcdh15 fragments was done in a stepwise manner as described previously [39], whereas cdh23 was dialyzed overnight [41]. Refolded proteins were further purified using size exclusion chromatography (SEC) on Superdex 75 or Superdex 200 16/600 columns (GE Healthcare) with SEC buffer containing 20 mM Tris-HCl pH 7.5, 150 mM KCl, 50 mM NaCl and 2 mM CaCl_2_. Purity of the recombinant proteins was analyzed by SDS–PAGE, after which the protein-containing fractions were pooled and used for further experiments. Predicted and apparent molecular weights (SDS–PAGE) for cdh23 and pcdh15 fragments were 23.8/25 kDa and 27.5/37 kDa, respectively, as previously observed [39].

The purification of tagless cdh23 fragments was done as described in the IMPACT kit protocol (NEB) with some modifications to improve yields. The constructs were expressed independently in BL21(DE3)-RIPL *E*. *coli* cells (Stratagene) cultured in LB medium and induced at OD ~ 0.6 with 400 μM IPTG at 15°C for ~16 h. Cells were lysed by sonication in denaturing buffer B. The cell lysate was centrifuged at 20,000 RPM for 30 min to remove cell debris. The clear lysate was first dialyzed for 24 h against regenerating buffer A (20 mM Tris-HCl pH 8.5, 0.5 M NaCl, 10 mM CaCl_2_) with 8 M urea, followed by two 24 h dialyses against regenerating buffer B and C with 6 M and 4 M urea, respectively. The last two steps consisted of 12 h dialyses against regenerating buffers D and E with decreasing urea concentration (2 and 0 M, respectively) plus 0.1 mM GSSG and 1 mM GSH. The dialyzed protein was centrifuged to remove any precipitate and affinity purified using chitin beads (NEB). Cleavage was induced using DTT for ~40 hours at 4°C. The cleaved cdh23 fragment was incubated with cleaned chitin beads to remove any traces of unbound intein and finally purified through gel filtration chromatography in SEC buffer. Protein purity was analyzed by SDS-page gel and protein-containing fractions were pooled accordingly.

### Designing high-affinity mutants

**Fig 2:**
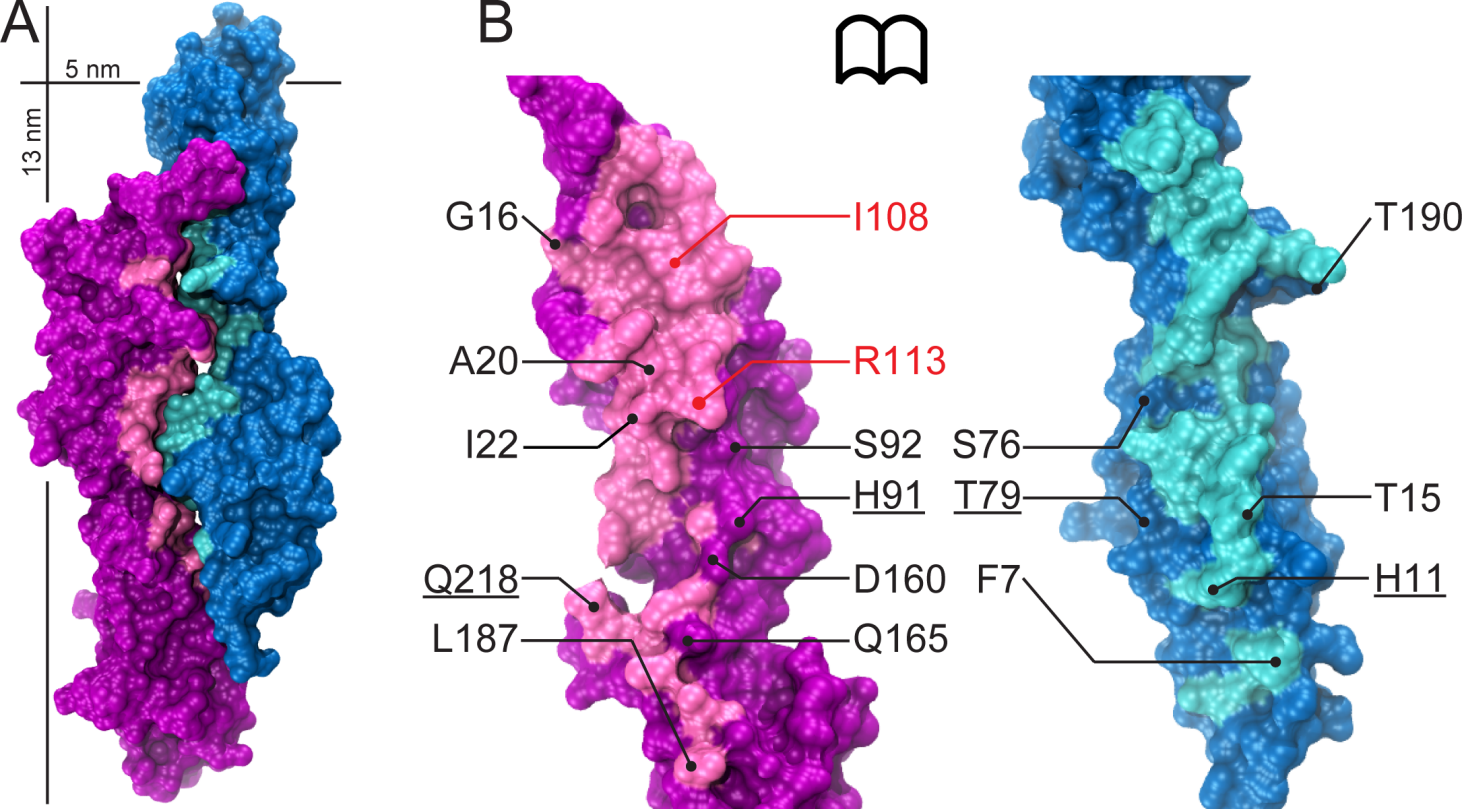
Mapping of rationally designed mutation sites on the structure of the cdh23 and pcdh15 complex. (A) Surface representation of cdh23 (blue and cyan) bound to pcdh15 (purple and pink; PDB ID: 4APX). (B) Cdh23 and pcdh15 interaction surfaces exposed with mutation sites labeled. Residues labeled in red are mutated in inherited deafness. Underlined labels indicate sites that belong to paired mutant complexes cdh23(H11K)-pcdh15(Q218E) and cdh23(T79E)-pcdh15(H91R).

We used the WT cdh23-pcdh15 complex structure (PDB ID: 4APX) to design mutations that enhance binding, since a number of stability and structural studies [42,43] show that point mutations usually have little effect on the overall structure of proteins except for the immediate vicinity of the mutation site. The interface was visually inspected and all possible sites of mutations were noted. *In silico* mutations were made using the mutate function of Coot [44]. All rotamers of the mutated amino acid were checked and *in vitro* site directed mutagenesis was performed only if over 50% of the favorable rotamers took the desired orientation (favoring salt-bridge formation or van der Waals interactions). The on-line webserver Rosetta design [45] was also used to find higher affinity mutants and the most stabilizing mutant (lowest *ΔΔG*) was picked for our experimental study (S1 Table). Co-crystallized ligands, alternate conformations of side chains, and water molecules were deleted during analysis with Rosetta design. All designed mutation sites (fourteen) for twelve complexes are shown on the surface of the protein complex in Fig 2, along with three mutation sites (I22, I108, and R113 in pcdh15) that were used for control experiments in which binding was impaired.

### Thermal scanning

Thermal scanning has been applied to the protein complex formed by off7 [46] and MBP (*K*_D_ ~4.4 nM at 25°C) as described in [26]. We modified this protocol to apply it to the inner-ear cdh23-pcdh15 complex (Fig 1B). The two proteins were mixed with the pcdh15 concentration fixed at 0.1 mg/mL (~4 μM) and the concentration of cdh23 increased from 0.1 to 0.5 mg/mL (~4 to 20 μM), thereby increasing the amount of complex formed at the beginning of the experiment according to Le Chatelier's principle. The 1:1 and 5:1 ratios of cdh23:pcdh15 correspond to ~75% and ~85% complex at *t* = 0 (*K*_D_ ~3 μM at *T* = 10°C), respectively. Solutions of 20 μL of protein sample per well were prepared by mixing 1 μL of 400x SYPRO Orange (Molecular Probes, final concentration 20x) with protein in SEC buffer and loaded into 96-well, 0.2 mL thin-wall PCR plates (BioRad) sealed with optical-quality sealing tape (BioRad). Thermal denaturation was performed using a CFX96 real time-PCR instrument (Bio-Rad) where temperature was increased in a step-wise manner from 10 to 95°C in 0.2°C/cycle increments (ramp rate of ~0.6°C/min) and with an equilibration time of 5 s at each temperature. Some control experiments used equilibration times of 20 or 51 s (S2 Table).

### Data fitting, estimation of melting temperatures, and statistical analysis

All thermal scanning data were analyzed in MATLAB (Mathworks). Melting curves were normalized and the temperature corresponding to a normalized fluorescence signal of 0.5 was defined as the melting temperature (*T*_m_). Derivative plots of fluorescence (change in fluorescence per unit change in temperature) were obtained from the slope of the normalized data as determined from a sliding five-point window around each temperature value. A student t-test was used to determine if selected temperature shifts (*ΔT*_m_, see Results) and dissociation constant (*K*_D_) differences were statistically significant. Data was imported to Excel and a two-tailed t-test (with unequal variance) was performed comparing each mutant complex to the WT complex. A p-value ≤ 0.05 was used as criterion for statistical significance.

### Surface plasmon resonance experiments

The interaction between cdh23 and pcdh15 was analyzed by SPR in a Biacore T100 instrument (GE Healthcare) with a Ni-NTA sensor chip (GE Healthcare). First, 0.5 mM NiCl_2_ was flowed at 10 μL/min on the chip for 60 s, followed by SEC buffer. The reference cell was prepared by immobilizing 6xHIS tagged pcdh15 on the sensor chip, using 1 to 1.5 μM of pcdh15 at a flow rate of 10 μL/min for 30 s, to obtain a surface density of >500 resonance units (RUs). Kinetic data were collected by injecting different concentrations of tag-free cdh23 ranging from 0.25 μM to 30 μM, diluted in SEC buffer, with a contact time of 60 s and a flow rate of 30 μL/min for both association and dissociation phases. After each measurement, the chip surface was regenerated with 500 mM imidazole at a flow rate of 30 μL/min for 90 s. All measurements were carried out at 25°C in SEC buffer filtered with 0.22 μm filters (Millipore). The *K*_D_ was calculated using the Biacore T100 evaluation software version 1.1. There was non-specific binding on the chip at high concentrations of cdh23 and thus those data points were neglected from the analysis. Also, any experiment with mass transport limitation was not included in the analysis. Kinetics analyses were performed using the EVILFIT software [47,48]. Briefly, the reference-subtracted sensorgram data were exported to Microsoft Excel, thereafter global analysis was conducted using a distribution model for continuous affinity. Before importing data into EVILFIT, the time column was multiplied by 10. This correction factor was later incorporated in the kinetic constant values. For fitting the data in EVILFIT, the high injection points were removed from analysis due to improper fittings at those concentrations. *K*_D_ values calculated by fitting of kinetic data using EVILFIT differed in magnitude with those obtained by fitting the hyperbolic concentration response curve (binding isotherms) in the Biacore evaluation software, likely due to fast association-dissociation rates that made EVILFIT fitting difficult. However, *K*_D_ trends were consistent regardless of the method used to compute them. We used *K*_D_ values obtained from binding isotherms to compare affinities among complexes.

### Analytical size exclusion chromatography

SEC experiments of all refolded proteins (WT and mutants of cdh23 and pcdh15) were performed on a Superdex 75 16/60 column with SEC buffer. Relevant fractions with pure protein fragments were collected, concentrated, and used for subsequent SEC on a Superdex 75 3.2/3.0 column equilibrated with the same buffer (S1 Fig). Experiments were performed at 4°C using a 100 μL loop and a 0.05 mL/min flow rate on an AKTAmicro system (GE).

### Circular dichroism spectroscopy

**Fig 3:**
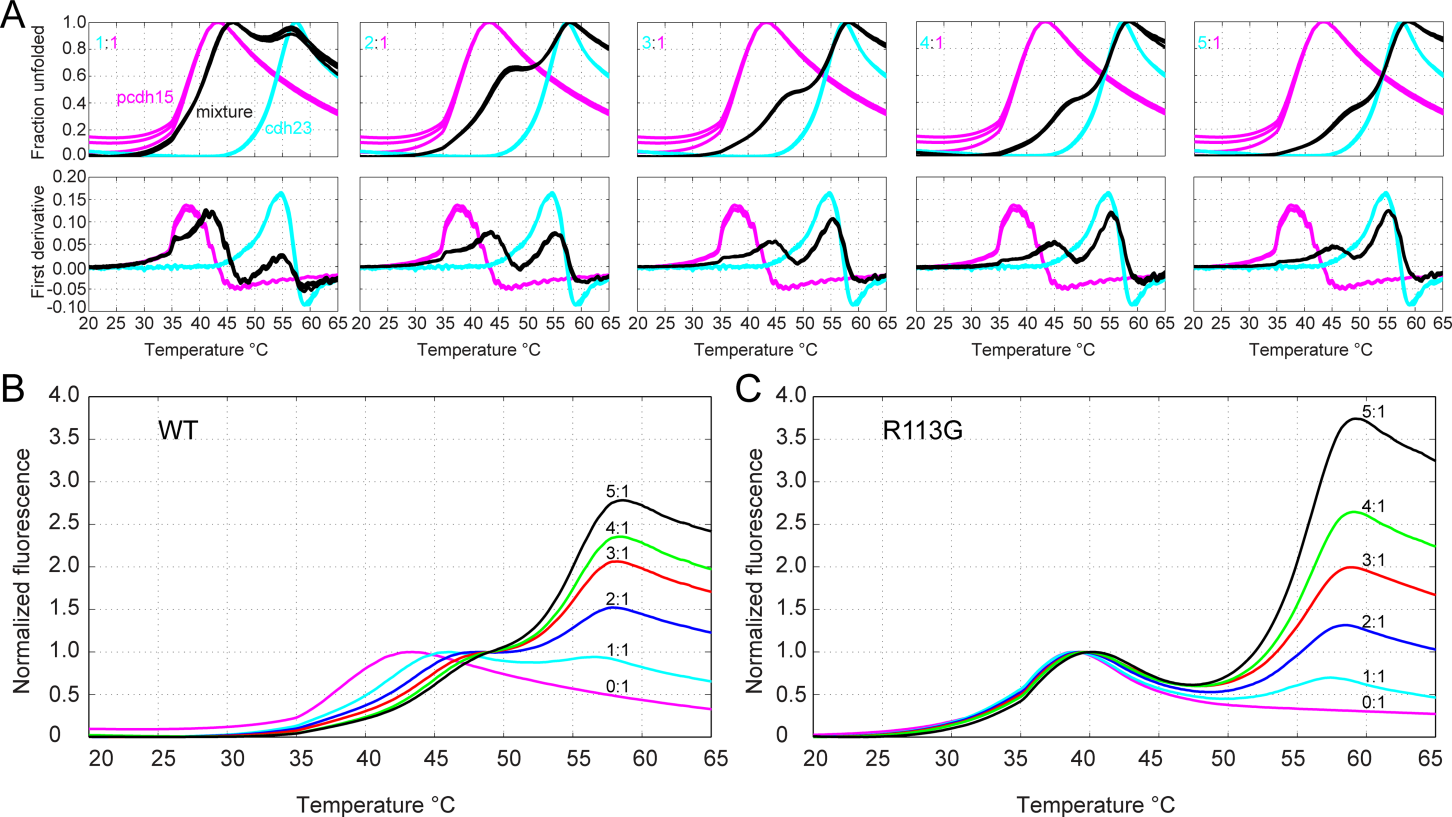
Thermal melting curves of cdh23 and pcdh15 variants monitored with SYPRO orange. (A) Upper panels show normalized fluorescence for melting curves with cdh23(WT) in cyan, pcdh15(WT) in magenta, and the mixture in black. Lower panels show the derivative of the normalized melting curve. Concentration ratios from left to right are: 1:1, 2:1, 3:1, 4:1, 5:1 (cdh23:pcdh15). (B-C) Overlay of the melting curves for pcdh15(WT) (B) and for pcdh15(R113G) (C) at indicated cdh23:pcdh15 ratios. The curves were normalized to pcdh15 only (first peak).

Spectra were obtained on a JASCO J-815 Circular Dichroism Spectrometer. Experiments were conducted at 20 μM concentration of pcdh15, determined by UV absorption (27,515 M^-1^ cm^-1^) at 280 nm, in SEC buffer (similar to experiments in [40]). Thermal unfolding and refolding spectra at 222 nm were acquired using a 0.4°C min^-1^ rate, with heating from 10 to 55°C and then cooling to 10°C (S3 Fig). We did not focus on cdh23 unfolding because it has a *T*_*m*_ > 50°C whereas all complexed WT or mutant pcdh15 proteins melt at lower temperatures (Fig 3A).

## Results

To study protein-protein interactions in a high-throughput fashion, we set out to investigate the application of thermal scanning assays using the CDH23-PCDH15 complex. We first analyzed the structure of the interacting tips of these proteins and identified mutations that may impair or enhance their binding. We then generated WT and mutant protein fragments, tested their integrity, and used thermal scanning experiments to detect complex formation, with additional quantification from SPR measurements as described below.

### Rational design of high-affinity mutant candidates

A number of methodologies have been developed to improve the binding affinity of protein-protein complexes. These include creating metal cation (like Ni^2+^) binding pockets at the interface [49,50], by extending the N-terminal region of one of the proteins to increase the interaction surface [51], through directed evolution [52,53], or by creating charged mutations near the interface [54]. We avoided the use of Ni^2+^ coordination in the protein complex as Ni^2+^ could interfere with Ca^2+^ binding to cadherin fragments [55]. Our cadherin fragments form inclusion bodies during bacterial expression thus preventing the use of standard bacterial-based directed evolution approaches. To overcome these issues, we used a simple, rational design approach to engineer the protein complex. We focused on the various non-covalent forces of attraction that are responsible for protein folding and protein-protein interactions—electrostatics, hydrogen bonds and van der Waals interactions. Then we used single or multiple mutations to enhance these interactions at the interface of our cadherin protein complex. The higher affinity mutant candidates were rationally planned with the goal of introducing salt bridges via charged amino acids at the interface, or with the purpose of burying more hydrophobic contacts through non-polar residues at the interface thereby increasing van der Waals interactions (Fig 2). The protein fragments carrying these designed mutations (or mutations known to impair complex formation) were used for experiments testing their integrity and ability to form complexes.

### Testing the integrity and interaction of cdh23 and pcdh15 mutants in solution

Analytical size exclusion chromatography (SEC) can be used to determine if cdh23 and pcdh15 fragments folded and form a complex. The individual WT fragments eluted as monodisperse peaks with well-defined elution volumes. WT complex formation was accompanied by a decrease in elution volume (*ΔEV*) of the mixture when compared to the elution volume of the individual protein fragments [39].

All mutants (shown in Fig 2) eluted as monodisperse peaks at the expected elution values for WT. The mutant pcdh15(A20V) did not form a complex as assessed by analytical SEC. Tests on three designed mutant complexes (cdh23(T15E)-pcdh15(G16D), cdh23(WT)-pcdh15(A20V), cdh23(T190W)-pcdh15(WT)) showed complex formation (S1 Fig), with varying *ΔEV* that might reflect changes in association rate constant *k*_on_ [56]. These experiments provided an initial, positive evaluation of the integrity of all mutants and an assessment of complex formation for some of them.

### Using thermal scanning to detect binding of cdh23 to pcdh15

Thermal stability shift assays have been used to probe the interaction between MBP and Off7 [26], and here we used similar protocols to probe interactions between the WT and mutant cdh23 and pcdh15 fragments tested using SEC as described above. These experiments required small amounts of label-free protein samples, could be set in a high-throughput format, were significantly faster than SEC, and had not been carried out with tip link cadherins.

Using thermal scanning we determined the melting temperature of pcdh15(WT), *T*_m_ = 37.9 ± 1.0°C, which increased in the presence of cdh23(WT) (Fig 3). This shift in *T*_m_, which we attribute to complex formation and denote as *ΔT*_m_, increased when the ratio of cdh23 to pcdh15 was increased from 1:1 to 5:1 (Fig 3A). By increasing the ratio of cdh23:pcdh15, we wanted to find the best non-stoichiometric ratio which we could use to compare *ΔT*_m_s of different mutants. We could not use non-stoichiometric ratios beyond 5:1, including the ratio 20:1 suggested in [26], as the fluorescence signal of cdh23 would overshadow that of pcdh15 in the complex (Fig 3A and 3B), impairing accurate determination of melting temperatures.

**Fig 4:**
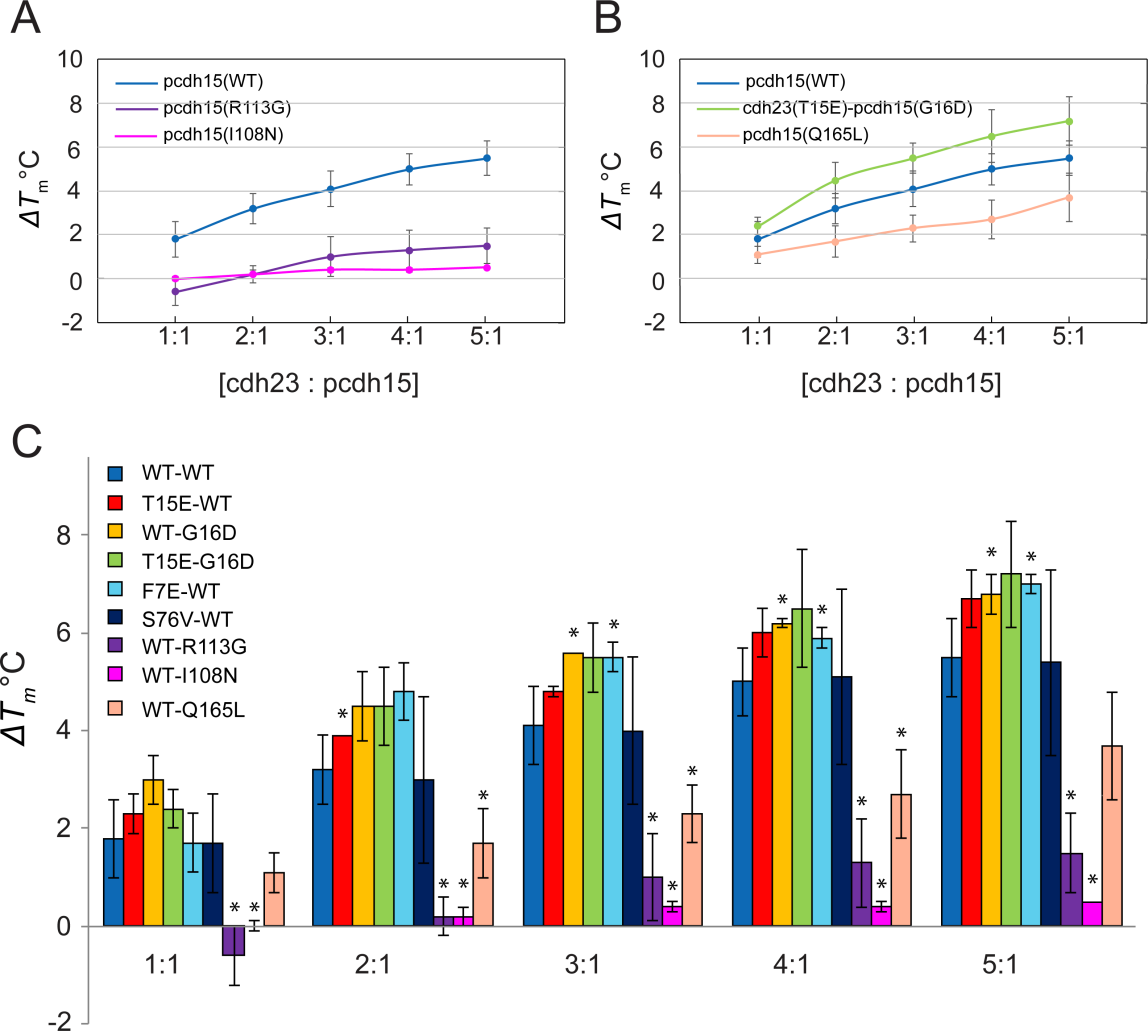
Variation of *ΔT*_m_ at increasing ratios of cdh23 and pcdh15. (A) Variation of *ΔT*_m_ for WT and two deafness-related complexes at increasing concentration ratios. The WT *ΔT*_m_ increases from ~2 to ~5.5°C from 1:1 to 5:1 ratios. The deafness mutants have *ΔT*_m_ < ~1°C at all ratios. (B) *ΔT*_m_ variation of the most stabilizing mutant G16D-T15E (green), the WT complex (blue), and the less stabilizing mutant Q165L (pink). The double mutant has higher *ΔT*_m_ than the WT complex at all ratios. (C) Bar graph of *ΔT*_m_ variation of selected mutants at different cdh23:pcdh15 ratios. Asterisks indicate statistically significant differences with WT complex (p ≤ 0.05). Data for all mutants including pcdh15- A20V, D160W, L187W, Q165L, S92V and cdh23-T190W are shown in Table 1.

To confirm that *T*_m_ shifts are caused by complex formation, we used a designed weak binder (I22A) and two deafness-related pcdh15 mutations (R113G and I108N) that impair binding to cdh23. The *T*_m_s of these pcdh15 mutants (I22A: 35.1 ± 0.1°C, R113G: 34.8 ± 0.0°C, I108N: 36.4 ± 0.2°C) were lower than for WT (37.9 ± 1.0°C), yet the protein fragments were stable enough to carry out the experiments (Table 1) and these weak affinity complexes served as controls. When the ratio of concentrations of cdh23 to pcdh15 was varied from 1:1 to 5:1, the *ΔT*_m_ for the cdh23(WT)-pcdh15(WT) complex ranged from 1.8 ± 0.8°C to 5.5 ± 0.8°C, whereas for the complex involving the deafness mutant pcdh15(R113G), the *ΔT*_m_ ranged from -0.6 ± 0.6°C to 1.5 ± 0.8 °C (Table 1 and Figs 3B, 3C and 4A). The failure of added cdh23 to raise the melting temperature of mutant pcdh15 suggests that it does not bind to the mutant. Similarly, for the complex involving the deafness mutant pcdh15(I108N), the *ΔT*_m_ barely varied from 0.0 ± 0.1°C to 0.5 ± 0.0°C. The *ΔT*_m_ values for the designed mutant pcdh15(I22A) were between those for the WT and those for the deafness mutants (R113G or I108N), consistent with the trend seen in previous studies [39,40]. Since the cdh23(WT) and pcdh15(R113G) and complex has a *K*_D_ > 20 μM [39], we attributed the low *ΔT*_m_ to lack of complex formation. The designed and deafness mutants served as controls and their very low *ΔT*_m_ correlated with their weak binding affinity (high *K*_D_). Overall, these thermal-scanning experiments allowed us to clearly identify when the interaction was impaired.

**Table 1:** Shift in melting temperature (*ΔT*_m_) for complexes of cdh23 bound to pcdh15. Deafness mutations are shown in bold. *ΔΔT*_m_ values are shown in parentheses.

Complex (cdh23:pcdh15)	*T*_m_ for pcdh15	*ΔT*_m_ at different cdh23:pcdh15 ratios					
		1:1	2:1	3:1	4:1	5:1	Predicted Improvement in affinity?
**WT:WT (n = 6)**	37.9 ± 1.0	1.8 ± 0.8	3.2 ± 0.7	4.1 ± 0.8	5.0 ± 0.7	5.5 ± 0.8	-
**T15E:WT (n = 2)**	37.9 ± 1.0	2.3 ± 0.4 (0.5)	3.9 ± 0.0 (0.7)	4.8 ± 0.1 (0.7)	6.0 ± 0.5 (1.0)	6.7 ± 0.6 (1.2)	Yes
**H11K:Q218E (n = 2)**	39.3 ± 1.8	1.2 ± 0.9 (-0.6)	2.3 ± 1.2 (-0.9)	3.4 ± 1.6 (-0.7)	4.0 ± 1.9 (-1.0)	4.4 ± 1.8 (-1.1)	No
**WT:G16D (n = 2)**	37.9 ± 1.4	3.0 ± 0.5 (1.2)	4.5 ± 0.7 (1.3)	5.6 ± 0.0 (1.5)	6.2 ± 0.1 (1.2)	6.8 ± 0.4 (1.3)	Yes
**T79E:H91R (n = 3)**	38.4 ± 0.9	2.1 ± 0.4 (0.3)	4.1 ± 0.5 (0.9)	5.3 ± 1.8 (1.2)	6.0 ± 1.8 (1.0)	7.3 ± 1.3 (1.8)	Yes
**F7E:WT (n = 2)**	37.9 ± 1.0	1.7 ± 0.6 (-0.1)	4.8 ± 0.6 (1.6)	5.5 ± 0.3 (1.4)	5.9 ± 0.2 (0.9)	7.0 ± 0.2 (1.5)	Yes
**T15E:G16D (n = 2)**	37.9 ± 1.4	2.4 ± 0.4 (0.6)	4.5 ± 0.8 (1.3)	5.5 ± 0.7 (1.4)	6.5 ± 1.2 (1.5)	7.2 ± 1.1 (1.7)	Yes
**WT:A20V (n = 2)**	38.5 ± 1.7	0.4 ± 0.0 (-1.4)	0.8 ± 0.4 (-2.4)	1.1 ± 0.4 (-3.0)	1.3 ± 0.3 (-3.7)	1.9 ± 0.7 (-3.6)	No
**WT:D160W (n = 2)**	35.5 ± 0.6	1.0 ± 0.1 (-0.8)	1.3 ± 0.5 (-1.9)	2.2 ± 0.2 (-1.9)	2.6 ± 0.3 (-2.4)	3.0 ± 0.3 (-2.5)	No
**WT:L187W (n = 3)**	38.7 ± 1.3	0.7 ± 0.4 (-1.1)	1.6 ± 0.7 (-1.6)	2.4 ± 1.0 (-1.7)	2.9 ± 1.2 (-2.1)	3.2 ± 0.8 (-2.3)	No
**WT:Q165L (n = 3)**	35.5 ± 0.2	1.1 ± 0.4 (-0.7)	1.7 ± 0.7 (-1.5)	2.3 ± 0.6 (-1.8)	2.7 ± 0.9 (-2.3)	3.7 ± 1.1 (-1.8)	No
**WT:S92V (n = 2)**	39.9 ± 0.6	0.7 ± 0.2 (-1.1)	1.1 ± 0.1 (-2.1)	1.7 ± 0.0 (-2.4)	2.4 ± 0.3 (-2.6)	3.0 ± 0.2 (-2.5)	No
**S76V:WT (n = 2)**	37.9 ± 1.0	1.7 ± 1.0 (-0.1)	3.0 ± 1.7 (-0.2)	4.0 ± 1.5 (-0.1)	5.1 ± 1.8 (0.1)	5.4 ± 1.9 (-0.1)	No
**T190W:WT (n = 2)**	37.9 ± 1.0	1.4 ± 0.2 (-0.4)	2.7 ± 0.6 (-0.5)	3.6 ± 0.8 (-0.5)	4.6 ± 1.2 (-0.4)	5.1 ± 1.1 (-0.4)	No
**WT:I22A (n = 2)**	35.1 ± 0.1	0.5 ± 0.2 (-1.3)	0.7 ± 0.0 (-2.5)	1.0 ± 0.1 (-3.1)	1.1 ± 0.1 (-3.9)	1.0 ± 0.3 (-4.5)	No
**WT:R113G (n = 3)**	34.8 ± 0.0	-0.6 ± 0.6 (-2.4)	0.2 ± 0.4 (-3.0)	1.0 ± 0.9 (-3.1)	1.3 ± 0.9 (-3.7)	1.5 ± 0.8 (-4.0)	No
**WT:I108N (n = 2)**	36.4 ± 0.2	0.0 ± 0.1 (-1.8)	0.2 ± 0.2 (-3.0)	0.4 ± 0.1 (-3.7)	0.4 ± 0.1 (-4.6)	0.5 ± 0.0 (-5.0)	No

### Thermal scanning assay identifies potential high-affinity complexes

To compare WT and mutant complexes, we defined *ΔΔT*_m_ as *ΔT*_m-mutant_ - *ΔT*_m-WT_ (where *ΔT*_m-mutant_ refers to *ΔT*_m_ of the mutant and *ΔT*_m-WT_ refers to *ΔT*_m_ of the WT) and used its value at high protein concentration ratios (4:1 or 5:1 for cdh23:pcdh15) to identify significant differences that might indicate impaired or enhanced interactions. A positive *ΔΔT*_m_ would indicate higher thermostability of the mutant complex, which could be correlated with higher binding affinity [26].

The designed mutant complexes, expected to have a higher affinity than the WT complex, showed a range of different *ΔΔT*_m_s, some being stabilizing while others being destabilizing (Table 1). The largest *ΔΔT*_m_ were observed in complexes involving non-polar/polar to charged residue mutations. Among the charged mutations, ~80% displayed higher *ΔT*_m_s (*ΔΔT*_m_ > 0) compared to the WT complex (cdh23(T15E), pcdh15(G16D), cdh23(T79E)-pcdh15(H91R), cdh23(F7E)), whereas all the mutations to non-polar/hydrophobic residues had *ΔΔT*_m_ < 0. The double mutant cdh23(T15E)-pcdh15(G16D) complex had *ΔΔT*_m_ values > 1°C at all ratios of cdh23:pcdh15 except 1:1 (Fig 4). Other mutants had *ΔΔT*_m_ values between those of the deafness mutants and the double mutant. If we extrapolate the trends in *ΔΔT*_m_ values from the 1:1 to 5:1 ratios used here to those required to obtain the *ΔΔT*_m_ at saturating concentrations (20:1 ratio of cdh23:pcdh15), a much higher value of *ΔΔT*_m_ would be obtained, suggesting that these mutants should have lower *ΔG* of binding [26].

Additional thermal scanning experiments with cdh23(WT)-pcdh15(WT) and cdh23(T15E)-pcdh15(G16D) were performed using slower temperature ramp rates in which the equilibration time (after a temperature increment and before taking a fluorescence measurement) was extended from 5 s to 20 s or 51 s (S2 Table). Results were consistent with those obtained using regular ramp rates.

### Surface plasmon resonance experiments test cdh23 and pcdh15 binding

**Fig 5:**
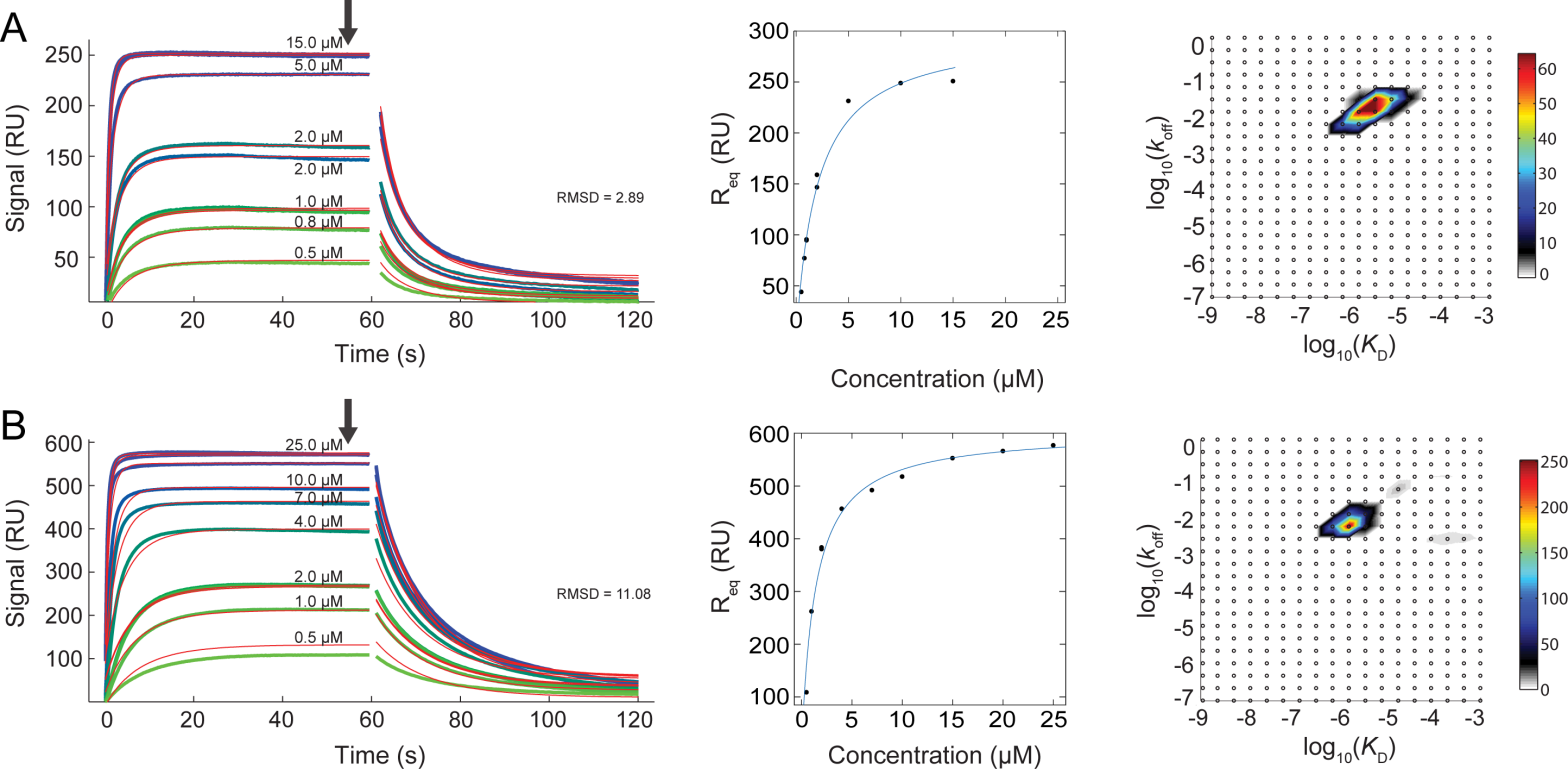
Quantitative SPR measurements of cdh23 molecules binding to pcdh15. (A) Left, association and dissociation of the cdh23(WT)-pcdh15(WT) complex. Experimental data (sensorgrams) are represented in a gradient of green to blue colors for different concentrations of cdh23(WT) as labeled. Red lines indicate fitted model parameters (RMSD = 2.89). Injection peaks were removed and not fitted. Top three traces correspond to 5, 10, and 15 μM, respectively, but the 10 μM trace is not labeled for clarity. Black arrow indicates the position of equilibrium SPR signal (R_eq_). Middle panel shows the fitting of R_eq_ to a Langmuir binding isotherm at different concentrations of analyte. Measurements for selected concentrations were done in duplicates. Right panel shows a heat map of the *k*_off_ and *K*_D_ distribution from the global fit of all traces in corresponding leftmost panel. The signal density of the peaks in the *k*_off_ and *K*_D_ distribution plot can directly be discerned from their color, which is scaled according to the color bar on the right side of the distribution plot [48]. (B) Association and dissociation curves for the T15E-G16D complex shown as in (A) (RMSD = 11.08). Top three traces correspond to 15, 20, and 25 μM, respectively, but the 15 and 20 μM traces are not labeled for clarity. The data were analyzed with the EVILFIT algorithm and the Biacore evaluation software.

To quantitatively compare *K*_D_ values for WT and mutant complexes formed by the cdh23 and pcdh15 fragments, we performed SPR experiments (Fig 5) with some of the complexes that displayed positive *ΔΔT*_m_ in thermal scanning experiments at the 5:1 ratio of cdh23:pcdh15. These included complexes formed by cdh23(T15E)-pcdh15(WT), cdh23(WT)-pcdh15(G16D), cdh23(T15E)-pcdh15(G16D) and cdh23(F7E)-pcdh15(WT). As a control, we also examined the mutant complex cdh23(WT)-pcdh15(Q165L) that had negative *ΔΔT*_m_ at all ratios, and cdh23(S76V)-pcdh15(WT) with nearly the same *ΔT*_m_ as the WT complex.

The *K*_D_ value for the cdh23(WT)-pcdh15(WT) complex was 1.38 ± 0.36 μM at 25°C (Table 2), consistent with isothermal titration calorimetry data (2.9 ± 0.4 μM at 10°C) [39]. Since this is an entropically driven reaction [39], a lower *K*_D_ value is expected at higher temperatures. The single-mutant complexes designed for higher affinity, cdh23(WT)-pcdh15(G16D) and cdh23(T15E)-pcdh15(WT), had *K*_D_ values of 1.11 ± 0.07 μM and 0.88 ± 0.06 μM respectively, which were lower than the WT complex. The double-mutant complex cdh23(T15E)-pcdh15(G16D) had the lowest *K*_D_ of 0.62 ± 0.11 μM among all the mutants that we tested (p-value 0.056). Surprisingly, a mutant complex with high *ΔΔT*_m_, cdh23(F7E)-pcdh15(WT), had *K*_D_ value similar to but not lower than that of the WT complex (1.58 ± 0.03 μM vs 1.38 ± 0.36 μM). On the other hand, a destabilized mutant complex cdh23(WT)-pcdh15(Q165L) had a *K*_D_ of 2.60 ± 0.0 μM, weaker than WT as expected from thermal scanning data. These results suggest that thermal scanning experiments can be used to screen for enhanced and impaired binding in a high-throughput, label-free manner, with some false positives.

**Table 2:** Affinity (*K*_D_) and kinetic parameters for complexes of cdh23 bound to pcdh15.

Complex (cdh23:pcdh15)	Biacore *K*_D_ (μM)	*k*_off_ (s^-1^)	*k*_on_ (M^-1^s^-1^) x 10^4^	*K*_D_ (μM)
**WT:WT (n = 3)**	1.38 ± 0.36	0.20 ± 0.04	6.19 ± 2.75	3.72 ± 1.65
**T15E:WT (n = 2)**	0.88 ± 0.06	0.11 ± 0.01	5.51 ± 0.85	2.05 ± 0.10
**WT:G16D (n = 2)**	1.11 ± 0.07	0.17 ± 0.01	9.48 ± 2.84	2.00 ± 0.99
**T15E:G16D (n = 2)**	0.62 ± 0.11	0.09 ± 0.00	6.14 ± 0.15	1.42 ± 0.05
**WT:Q165L (n = 2)**	2.60 ± 0.00	0.22 ± 0.04	5.59 ± 0.42	3.94 ± 1.11
**S76V:WT (n = 3)**	1.16 ± 0.56	0.18 ± 0.01	6.88 ± 2.28	2.79 ± 0.88
**F7E:WT (n = 2)**	1.58 ± 0.03	0.22 ± 0.04	6.60 ± 0.71	3.25 ± 0.25

**Fig 6:**
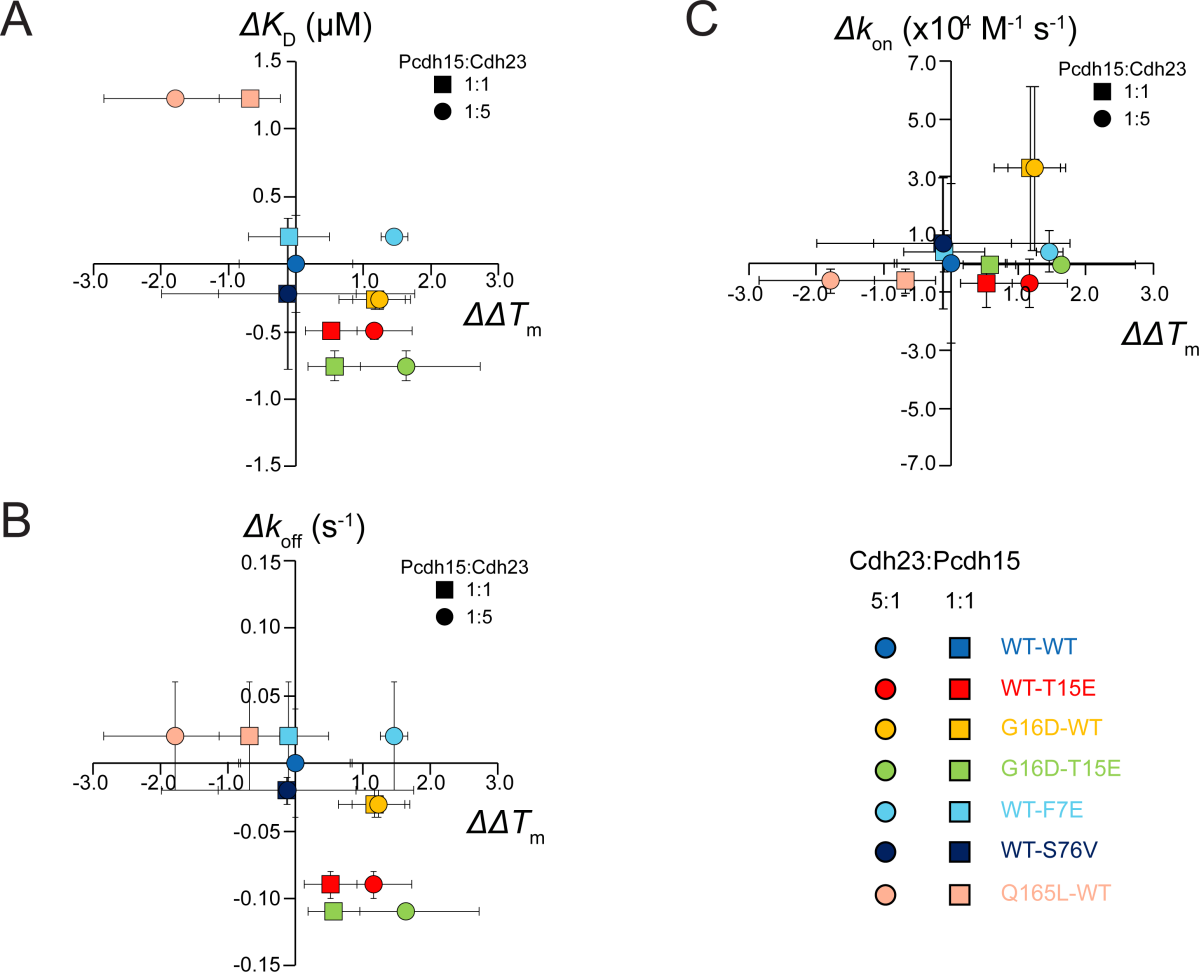
Rate and equilibrium constants for different mutants vs *ΔΔT*_m_. (A-C) *K*_D_ vs *ΔΔT*_m_ (A), *k*_off_ vs *ΔΔT*_m_ (B), and *k*_on_ vs *ΔΔT*_m_ (C) for various cdh23-pcdh15 complexes (WT-WT: light blue; T15E-WT: red; WT-G16D: yellow; T15E-G16D: light green, F7E-WT: cyan; S76V-WT: navy blue; WT-Q165L: pink). Squares and circles represent data points for *ΔΔT*_m_ at 1:1 and 5:1 ratios, respectively. The WT-WT complex lies at the origin. Data points along the dashed diagonal line in A support *ΔΔT*_m_ as a good predictor of *K*_D_, while data points off the diagonal indicate exceptions (most notably F7E-WT). Vertical error bars represent standard deviation for measurements of the rate or equilibrium constant of that mutant. Horizontal error bars represent the standard deviation for the *ΔT*_m_ measurement.

To qualitatively understand the origin of the differences between thermal scanning and SPR measurements, we looked at correlations between dissociation constants (*K*_D_), kinetic rate constants (*k*_off_ and *k*_on_) and *ΔΔT*_m_. Plots of *ΔK*_D_, *Δk*_off_ and *Δk*_on_ as a function of *ΔΔT*_m_ for multiple mutant complexes (Fig 6) revealed qualitative correlations between *ΔΔT*_m_ with the dissociation rate *k*_off_, and the affinity *K*_D,_ but not with *k*_on_. These results suggest that *ΔΔT*_m_ can be used to detect changes in *k*_off_, and only in some cases changes in *K*_D_.

## Discussion and conclusions

Methods that can characterize protein-protein interactions in a high throughput fashion are essential in the quest to find mutations or small molecules that may impair or enhance the formation of biomolecular complexes. Here we implemented a thermal scanning methodology to test tip-to-tip interactions of CDH23 and PCDH15, two proteins essential for inner-ear mechanotransduction. This assay allowed us to readily detect the formation of the WT cdh23 and pcdh15 complex by monitoring melting temperature shifts, which were absent when pcdh15 carried deafness mutations known to impair interactions with cdh23. In addition, we used this assay to evaluate rationally designed mutations predicted to enhance binding of pcdh15 to cdh23, and found a double mutant complex (cdh23(T15E)-pcdh15(G16D)) in which binding affinity was enhanced by 1.98 kJ/mol as determined by SPR measurements. Overall, this method produced some false positives in screening for enhanced binding, but robustly detected impaired complex formation.

A thorough quantitative analysis of thermal scanning results for another protein system was performed in [26], where a thermodynamic model was used to analyze the complex formed by the MBP and off7 proteins. The model assumed that binding interactions occur only between native states and that unfolding transitions for the lower melting partner were reversible, as shown for MBP [57]. This is not valid in our case as the unfolding of pcdh15 is irreversible (S2 and S3 Figs), which limits quantitation. For a fully irreversible system, we would expect the changes in *T*_m_ to reflect only the changes in the rate of unfolding, which itself will be affected by the rate of dissociation, but actually the *T*_m_ shifts correlate better to some degree with both *K*_D_ and *k*_off_ values. This suggests that the basis of the ability to detect binding differences is that the dissociation and unfolding are partially reversible at least very near the melting transition. Indeed, even for MBP, reversibility is only observed close to the melting transition in the thermal scanning experiment. The kinetic effects of partial irreversibility may explain some of the false-positives in our predictions of complexes with enhanced affinity from the thermal scanning data.

The ability to quickly detect impaired pcdh15 and cdh23 binding might help evaluate and understand the structural effects of new mutations involved in inherited deafness. There are over one hundred missense mutations that modify the extracellular domain of CDH23 and that are considered to be the cause of a deafness phenotype [58–60]. Similarly, a handful of missense mutations involved in deafness modify the extracellular domain of protocadherin-15 [37,40,61]. Here, we tested three mutations (R113G, I108N, I22A) in the EC1+2 repeats of PCDH15 known to impair complex formation as evaluated using other methods, including isothermal titration calorimetry [34,38,39]. Two of these mutations (R113G, I108N) cause deafness, while the third one (I22A) has not been evaluated in animal models. In all cases, we could readily detect the lack of binding of the mutant pcdh15 to cdh23(WT). As new variations in human *CDH23* and *PCDH15* genes are discovered, this *in vitro* test may help to quickly determine the degree to which they impair binding and thus whether they should be presumed pathogenic. The assay may also be used to perform unbiased screens of mutations that critically affect the interface, perhaps indirectly, to further understand binding specificity and the structural determinants of tip link assembly.

Our assay is ideally suited to carry out screens to search for small-molecules inhibitors of the CDH23 and PCDH15 interaction. Disruption of the cdh23 and pcdh15 complex is known to impair tip-link function and inner-ear mechanotransduction. However, transient block of this interaction using small molecules might be helpful in two ways. First, elegant experiments have used calcium chelators such as BAPTA to disrupt tip-link cadherins, eliminate tip links in hair cells, and then study subsequent *ex vivo* tip-link regeneration upon restoration of physiological calcium levels [33,62,63]. This type of experiment likely recapitulates tip link assembly during development, and may also model tip-link regeneration after loud sound exposure [38,62,64,65]. Unfortunately, the use of calcium chelators may also alter various calcium-signaling pathways important for hair-cell function, so uncoupling of tip-link disruption from calcium chelation is desirable. Second, some antibiotics and cancer drugs that likely enter hair cells through their transduction machinery cause apoptosis and hearing loss [66–70]. Transient block of tip links might help investigate these phenomena to perhaps develop strategies that prevent hair-cell damage during these treatments. The methods presented here should enable the search for small-molecule blockers that could be used to understand tip-link regeneration and the effect of ototoxic drugs on hair-cell survival.

Finding mutations and small molecules that enhance binding affinity for the cdh23 and pcdh15 complex could also be enabled by our assay, as illustrated by the cdh23(T15E)-pcdh15(G16D) complex with increased affinity. A stronger tip link may result in more robust hair-cell responses to mechanical stimuli and prevention of progressive hearing loss. A similar approach could be applied to search for small-molecule candidates that can restore impaired binding in deafness mutants of cdh23 and pcdh15 [34,39,40].

The thermal scanning method presented here could also be used to quickly test interactions among other members of the cadherin superfamily of proteins. There are over one hundred cadherins encoded in the human genome, many of which form adhesive complexes involved in various essential physiological processes. However, the method would have to be extended and tested with protein homodimers, as most members of the cadherin superfamily form homophilic complexes. In addition, this method might be suitable for other protein complexes and can be easily extended for high-throughput screening of libraries of therapeutically relevant small molecules to find inhibitors of protein-protein interactions.

## Supporting information

S1 Table Results of Rosetta design are shown. The predicted high affinity pcdh15 mutations are marked in bold. Mutations with *ΔΔG*_binding_ > -0.5 Kcal/mol are not included.
(DOCX)

S2 Table Control thermal scanning experiments performed at slower ramp rates (increased equilibration time). The values of *ΔT*_m_ at different cdh23:pcdh15 ratios are shown for the WT-WT and T15E-G16D complex. These experiments were performed with an equilibration time (τ) of 20 s and 51 s (as opposed to 5 s used in our standard thermal scanning experiment). Values given in parentheses represent experiment performed with an equilibration time of 51 s. The *ΔT*_m_ marked with an asterisk was outside μ ± 2σ (where μ represents mean and σ represents standard deviation) of our regular thermal scanning experiments.
(DOCX)

S1 Fig Analytical SEC experiments of complex formation. Representative curves of WT and selected cdh23 and pcdh15 mutants. Decrease in elution volume is representative of complex formation. **(A)** The cdh23(T15E)-pcdh15(G16D) complex peak elutes at nearly the same place as the WT complex (green). **(B)** Shift in elution volume for the mutant mixture of cdh23(WT)-pcdh15(A20V) is less than that for the WT mixture. **(C)** Deafness mutant pcdh15(I108N) does not form a complex with cdh23(WT). As a result, the SEC trace of cdh23(WT)-pcdh15(I108N) mixture is the sum of individual traces of cdh23(WT) and pcdh15(I108N). **(D)** The cdh23(T191W) mutant forms a complex with pcdh15(WT) and has a shift similar to that of the WT complex.
(TIF)

S2 Fig Irreversible unfolding of pcdh15 revealed by circular dichroism (CD) spectroscopy. To determine whether the unfolding of pcdh15 is reversible or irreversible, we performed circular dichroism (CD) measurements in which the WT protein was heated and subsequently cooled down. The unfolding and refolding followed a clear hysteresis and once denatured, pcdh15 did not seem to fold upon cooling (S3 Fig). We performed the experiment with two different waiting times and observed similar behavior. The *T*_m_ calculated from CD was 41.3°C, slightly higher than the value obtained from thermal scanning experiments. The *T*_m_ recorded from these two techniques has been shown to be roughly related in a linear fashion and the offset between the two depends on buffer and dye conditions [22]. These data indicate that the interpretation of thermal scanning results must incorporate irreversibility of unfolding of pcdh15, as illustrated in the scheme below.
(DOCX)

S3 Fig Circular dichroism experiment to determine reversibility of unfolding of pcdh15. **(A)** Full CD trace of pcdh15(WT) at 222 nm monitored while heating from 10 to 55°C (filled circles) and cooling back 10°C (open circles) with a ramp rate of 0.4°C/min. Black and blue circles represent data points with wait times (before recording the measurement) of 6 s and 12 s, respectively. Data were normalized to the range of the melting curve. **(B)** CD spectra of the folded pcdh15(WT). Unfolding of pcdh15(WT) is irreversible.
(TIF)
