# Supplementary material for: Using thermal scanning assays to test protein-protein interactions of inner-ear cadherins
Source: PLoS One. 2017 Dec 19;12(12):e0189546. doi: 10.1371/journal.pone.0189546 (PMC5736220; doi:10.1371/journal.pone.0189546)
Supplement: S1 Table — The predicted high affinity pcdh15 mutations are marked in bold. Mutations with ΔΔGbinding > -0.5 Kcal/mol are not included. (DOCX) [file pone.0189546.s001.docx]

# **S1 Table.** Results of Rosetta Design are shown. The predicted high affinity pcdh15 mutations are marked in bold. Mutations with *ΔΔG*_binding_ > -0.5 Kcal/mol are not included.

| Mutation | *ΔΔG*_binding_ (Kcal/mol) | *ΔΔG*_cdh23_ (Kcal/mol) | *ΔΔG*_pcdh15_ (Kcal/mol) |
| --- | --- | --- | --- |
| L187W | -1.8 | 0.0 | 1.0 |
| V2F | -0.9 | -0.1 | 0.0 |
| V2Y | -0.9 | -0.1 | 0.0 |
| V2W | -0.6 | 0.2 | 0.0 |
| R117M | -0.5 | 0.0 | -1.9 |
