# Supplementary material for: Using thermal scanning assays to test protein-protein interactions of inner-ear cadherins
Source: PLoS One. 2017 Dec 19;12(12):e0189546. doi: 10.1371/journal.pone.0189546 (PMC5736220; doi:10.1371/journal.pone.0189546)
Supplement: S2 Table — The values of ΔTm at different cdh23:pcdh15 ratios are shown for the WT-WT and T15E-G16D complex. These experiments were performed with an equilibration time (τ) of 20 s and 51 s (as opposed to 5 s used in our standard thermal scanning experiment). Values given in parentheses represent experiment performed with an equilibration time of 51 s. The ΔTm marked with an asterisk was outside μ ± 2σ (where μ represents mean and σ represents standard deviation) of our regular thermal scanning experiments. (DOCX) [file pone.0189546.s002.docx]

# **S2 Table.** Control thermal scanning experiments performed at slower ramp rates (increased equilibration time). The values of *ΔT*_m_ at different cdh23:pcdh15 ratios are shown for the WT-WT and T15E-G16D complex. These experiments were performed with an equilibration time (τ) of 20 s and 51 s (as opposed to 5 s used in our standard thermal scanning experiment). Values given in parentheses represent experiment performed with an equilibration time of 51 s. The *ΔT*_m_ marked with an asterisk was outside µ ± 2σ (where µ represents mean and σ represents standard deviation) of our regular thermal scanning experiments.

| cdh23:pcdh15 | *ΔT*_m_ cdh23(WT)-pcdh15(WT) | | *ΔT*_m_ cdh23(T15E)-pcdh15(G16D) | |
| --- | --- | --- | --- | --- |
|  | τ **= 20 s** | τ = **51 s** | τ **= 20 s** | τ **= 51 s** |
| 1:1 | **1.3** | **1.8** | **3.0** | **3.7*** |
| 2:1 | **2.8** | **2.9** | **4.8** | **5.1** |
| 3:1 | **3.4** | **3.5** | **6.0** | **6.7** |
| 4:1 | **3.7** | **4.5** | **6.7** | **7.1** |
| 5:1 | **4.4** | **6.2** | **7.1** | **7.5** |
