# Supplementary figures and images for: Using thermal scanning assays to test protein-protein interactions of inner-ear cadherins
Source: PLoS One. 2017 Dec 19;12(12):e0189546. doi: 10.1371/journal.pone.0189546 (PMC5736220; doi:10.1371/journal.pone.0189546)

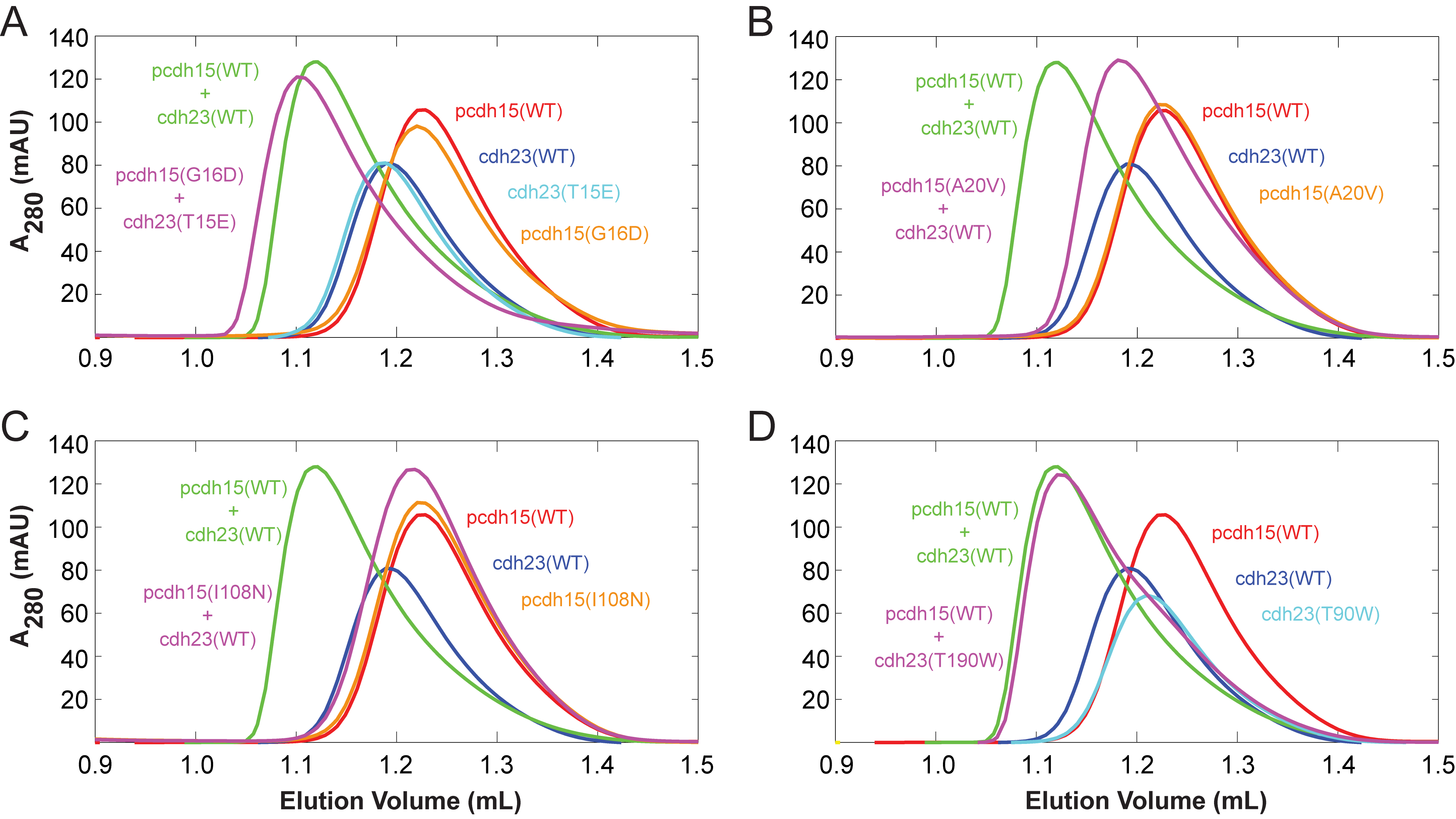

Supplement: S1 Fig — Representative curves of WT and selected cdh23 and pcdh15 mutants. Decrease in elution volume is representative of complex formation. (A) The cdh23(T15E)-pcdh15(G16D) complex peak elutes at nearly the same place as the WT complex (green). (B) Shift in elution volume for the mutant mixture of cdh23(WT)-pcdh15(A20V) is less than that for the WT mixture. (C) Deafness mutant pcdh15(I108N) does not form a complex with cdh23(WT). As a result, the SEC trace of cdh23(WT)-pcdh15(I108N) mixture is the sum of individual traces of cdh23(WT) and pcdh15(I108N). (D) The cdh23(T191W) mutant forms a complex with pcdh15(WT) and has a shift similar to that of the WT complex. (TIF) [file pone.0189546.s003.tif]

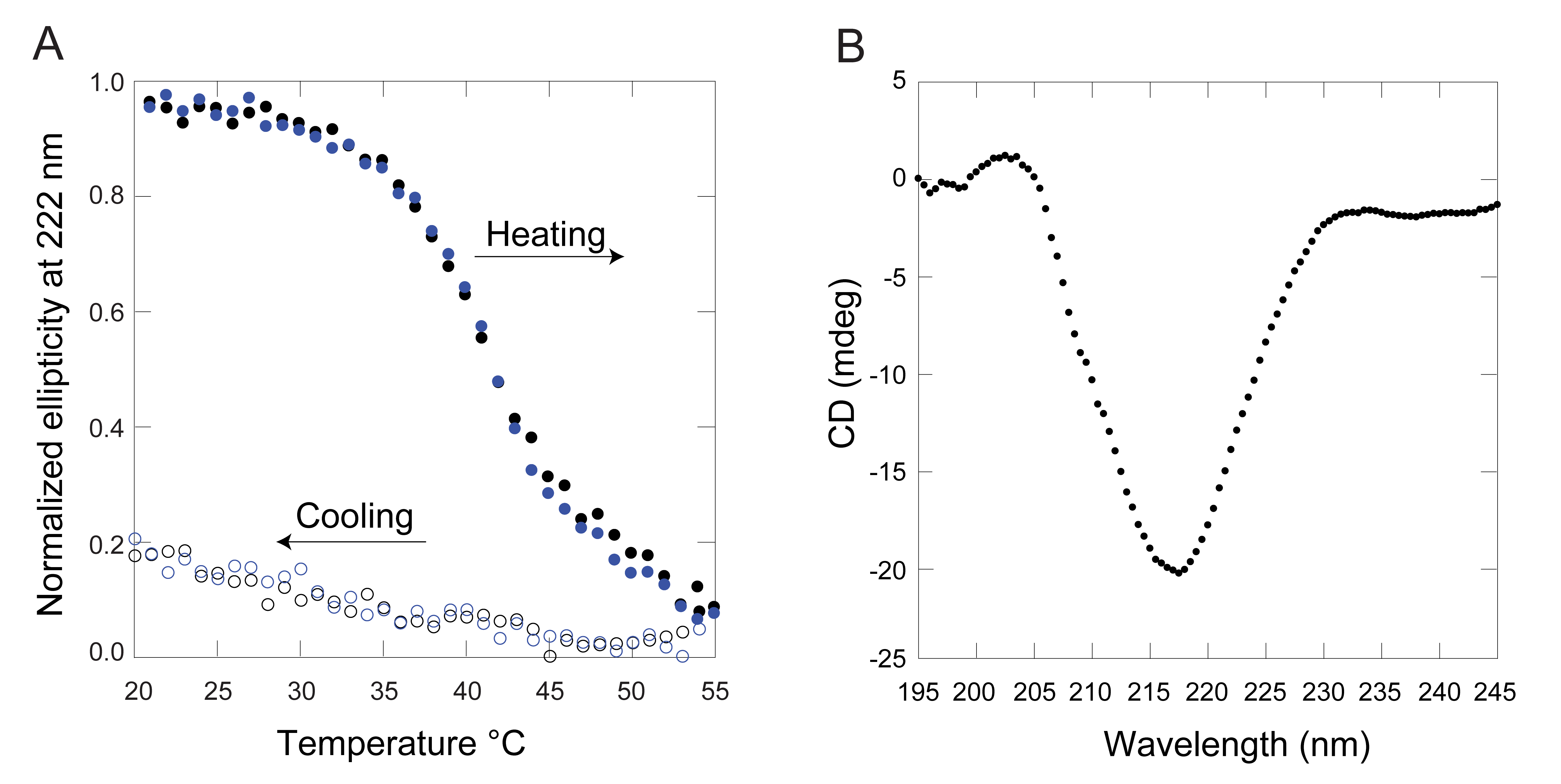

Supplement: S3 Fig — (A) Full CD trace of pcdh15(WT) at 222 nm monitored while heating from 10 to 55°C (filled circles) and cooling back 10°C (open circles) with a ramp rate of 0.4°C/min. Black and blue circles represent data points with wait times (before recording the measurement) of 6 s and 12 s, respectively. Data were normalized to the range of the melting curve. (B) CD spectra of the folded pcdh15(WT). Unfolding of pcdh15(WT) is irreversible. (TIF) [file pone.0189546.s005.tif]
