# Supplementary material for: Using thermal scanning assays to test protein-protein interactions of inner-ear cadherins
Source: PLoS One. 2017 Dec 19;12(12):e0189546. doi: 10.1371/journal.pone.0189546 (PMC5736220; doi:10.1371/journal.pone.0189546)

**S2 Fig. Irreversible unfolding of pcdh15 revealed by Circular Dichroism (CD) spectroscopy.**

To determine whether the unfolding of pcdh15 is reversible or irreversible, we performed circular dichroism (CD) measurements in which the WT protein was heated and subsequently cooled down. The unfolding and refolding followed a clear hysteresis and once denatured, pcdh15 did not seem to fold upon cooling (S3 Fig). We performed the experiment with two different waiting times and observed similar behavior. The *T*_m_ calculated from CD was 41.3 °C, slightly higher than the value obtained from thermal scanning experiments. The *T*_m_ recorded from these two techniques has been shown to be roughly related in a linear fashion and the offset between the two depends on buffer and dye conditions [22]. These data indicate that the interpretation of thermal scanning results must incorporate irreversibility of unfolding of pcdh15, as illustrated in the scheme below.


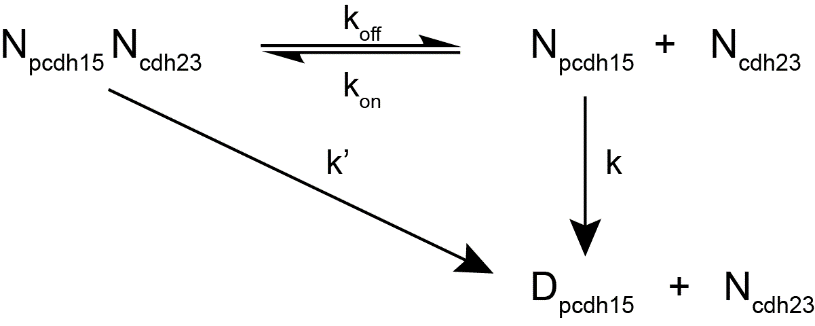

Supplement: S2 Fig — To determine whether the unfolding of pcdh15 is reversible or irreversible, we performed circular dichroism (CD) measurements in which the WT protein was heated and subsequently cooled down. The unfolding and refolding followed a clear hysteresis and once denatured, pcdh15 did not seem to fold upon cooling (S3 Fig). We performed the experiment with two different waiting times and observed similar behavior. The Tm calculated from CD was 41.3°C, slightly higher than the value obtained from thermal scanning experiments. The Tm recorded from these two techniques has been shown to be roughly related in a linear fashion and the offset between the two depends on buffer and dye conditions [22]. These data indicate that the interpretation of thermal scanning results must incorporate irreversibility of unfolding of pcdh15, as illustrated in the scheme below. (DOCX) [file pone.0189546.s004.docx]
